# Green synthesis of ZnO nanoparticles decorated on polyindole functionalized-MCNTs and used as anode material for enzymatic biofuel cell applications

**DOI:** 10.1038/s41598-020-61831-4

**Published:** 2020-03-19

**Authors:** Inamuddin, Nimra Shakeel, Mohd Imran Ahamed, Suvardhan Kanchi, Heba Abbas Kashmery

**Affiliations:** 1 Chemistry Department, Faculty of Science, King Abdulaziz University, P. O. Box 80203, Jeddah, 21589 Saudi Arabia; 2 Department of Chemistry, Faculty of Science, Aligarh Muslim University, Aligarh 202002 Uttar Pradesh, India; 3 Department of Chemistry, Faculty of Applied Science, Durban University of Technology, Durban, 4000 South Africa

**Keywords:** Energy, Fuel cells

## Abstract

Presently, one of the most important aspects for the development of enzymatic biofuel cells (EBFCs) is to synthesize the novel electrode materials that possess high current density, low open-circuit voltage (OCV) and long-term stability. To achieve the above attributes, lots of new strategies are being used by the researchers for the development of advanced materials. Nowadays, nanomaterials and nanocomposites are the promising material that has been utilized as effective electrode material in solar cells, supercapacitors and biofuel cells application. Herein, we account for a novel electrocatalyst as electrode material that comprised ZnO nanoparticles decorated on the surface of polyindole (PIn)-multi-walled carbon nanotube (MWCNT), for the immobilization of glucose oxidase (GOx) enzyme and mediator (Ferritin). The PIn-MWCNT scaffold is prepared via *in situ* chemical oxidative polymerization of indole on the surface of MWCNT and assessed by myriad techniques. The micrograph of scanning electron microscopy (SEM) designated the interconnected morphology of MWCNTs in the polymer matrix. X-ray diffraction spectroscopy (XRD) and Fourier transform infrared spectroscopy (FTIR), confirm the crystallinity and different functional groups available in the synthesized material, respectively. The electrochemical assessment demonstrates that the ZnO/PIn-MWCNT/Frt/GOx nanobiocatalyst exhibits much higher electrocatalytic activity towards the oxidation of glucose with a maximum current density of 4.9 mA cm^−2^ by consuming 50 mM glucose concentration in phosphate buffer saline (PBS) (pH 7.4) as the testing solution by applying 100 mVs^−1^ scan rates. The outcomes reflect that the as-prepared ZnO/PIn-MWCNTs/Frt/GOx biocomposite is a promising bioanode for the development of EBFCs.

## Introduction

Recently, enzymatic biofuel cell (EBFC) is a modern green renewable technology that can harness hidden electrical energy present in the chemical bonds of the replicate fuels, while catalyzing them with the assistance of redox enzymes as the electrocatalyst^1^. EBFCs are exploiting a large number of biological fuels such as lactate^2^, fructose^3^, starch^4^, and glucose^5^. Moreover, favorable operating conditions like ambient temperature^6^, mild pH^7^, and use of biological catalysts make EBFCs dominant over the traditional fuel cell, which in contrast, used noble metal catalysts (Platinum, gold, and silver), variable pH and working temperature range. Because of these characteristics, EBFCs has received immense attention as power generators in portable devices and automobiles^8^.

Glucose oxidase (GOx) is a commonly utilized enzyme in EBFCs, owing to possess specificity towards the glucose fuel that is abundantly available as biomass in the surrounding as well as in the physiological fluid. Fascinatingly, utilization of biofuels, particularly glucose, has drawn special attention in powering various implantable medical electronic devices such as pacemakers^9^, miniaturized sensors^5^, transmitters^10^, and artificial organs^11^ due to the occurrence of glucose in the living fluid. Moreover, the advantage of using biocatalyst gains the specificity towards a particular substrate which circumvent the need of membrane and allows the simplification in architecture as a membrane-less setup.

Although the significant outcomes have been attained, further advancements are still looked-for, such as low power/current densities, high open-circuit voltage (OCV), and instability over time for complete realization in a real-world application. These hurdles are directly related to inefficient electron transfer between the enzyme active site to the electrode, which is directly associated with the poor immobilization of enzyme at the electrode surface.

Currently, various strategies have been used to enhance the performance of EBFCs. One is the direct electron transfer (DET)^12^ whereby electrode modified with satisfactory group on nanoscale materials to improve the surface infrastructure to transfer electrons directly from the enzyme active sites to the electrode surface; on the other side mediator is used where electrons shuttle with the aid of redox mediator namely mediated electron transfer (MET)^13^. Generally, DET is more extensively preferred over the MET because it circumvents all the downsides associated with a mediator, for instance, leakage that can influence the biological activity of enzymes and thereby able to affects the kinetics of the electrochemical reaction. Nevertheless, direct electrochemistry of GOx is still a problem, as the position of redox-active center FAD is deeply situated, which resists the electron shuttling by expanding the path of electron communication^14^. To tackle these issues, redox-active mediators have been introduced. Up to now, a large number of redox-active mediators have been reported in the literature^15–17^. Ferritin (Frt) is an eco-friendly, biocompatible, and biodegradable redox protein that has the capability of holding iron molecules during the redox catalyzed reaction. Because of these attributes, it has been utilized by glucose-based EBFCs^18–20^. One of the foremost advantages in selecting Frt is that it has working potential close to the GOx enzyme that prevents ohmic loss during the electron transfer reactions and maintains the kinetics of bio-catalyzed reactions.

Recently, conductive materials especially carbon nanomaterials such as graphene^21^ and carbon nanotube (CNTs)^22^, have been extensively used as supporting catalyst in fuel cell applications not only due to their unique morphologies such as high specific surface area but they also show exciting properties as a corrosion resistance, good electronic conductivity, and high stability. In particular, carbon nanotubes (CNT) make a boom in the field of electrical devices due to their dimension (high aspect ratio and high specific surface area), high electronic conductivity, and excellent mechanical and chemical properties^23^. The high conductivity of CNTs is due to the availability of free electrons owing to the sp^2^ hybridized carbon atoms of the hexagonal graphitic plane. Along with these attributes, CNTs placed as privileged material in the field of biofuel cells^24,25^. Moreover, from the literature, it is noted that CNT decorated nanoparticles exhibit more catalytic activity than the pristine CNTs^26^. Surprisingly, Pt-decorated on the walls of CNTs delivered a high catalytic performance in methanol oxidation reaction (MOR)^27^, dispersed gold nanoparticles on CNTs is good immunosensor as the pristine one^28^ where as Co-doped Ni_3_S_2_@CNT arrays anchored on carbon nanotubes delivered more hydrogen evolution compared to the pristine one^29^.

Along with these nanoparticles (NPs), incorporation of zinc oxide nanoparticles (ZnO NPs) on the surface of carbon nanotubes (CNTs) has been exploited in colorimetric detection of cholesterol^30^, whereas, zinc oxide nanoparticles incorporated graphene–carbon nanotubes showed high electrocatalytic ability towards glucose detection^31^. All these exploitations of ZnO-NPs are because of their unusual properties, i.e., the high surface area for strong adsorption of biomolecules, good biocompatibility, chemical stability, non-toxicity, and high electron communication^32^. Moreover, the green synthesized ZnO NPs eliminate the use of toxic chemicals that employed biological extract (Neem leaves extract) as reducing and stabilizing agents that further improved biocompatibility^33^. Though the distribution, deposition, and morphology of NPs reinforced by CNTs strongly depend on the surface properties of the CNTs. As the pristine CNTs are chemically inert and do not have adequate sites for binding nanoparticles, which in turn poor scattering and accumulation of nanoparticles. So, CNTs functionalized by conducting polymers have received immense attention that develops a strong communication link with the nanoparticles and CNTs while keeping the intrinsic properties of them untouched. Also, there are various conducting polymers which form the especial combination with the unique properties of CNTs and developed the multifunctional materials with immense potential and have been explored in large number of fields^34^. For instance polyaniline-grafted CNTs as electrode materials for supercapacitors^35^, polypyrrole coated CNTs as a gas sensor^36^, and CNT/PEDOT transparent conductive films as solar cell^37^ etc. It is to be well noted that including various polymer, to date polyindole (PIn) has received immense attention due to its good electrical properties, environmental stability, and ease of synthesis^38^. Including this PIn has both benzene and pyrrole rings that reflect the properties of two, for instance high storage ability, good thermal stability, along with slow degradation rate as compared to the Ppy and PANI^39^. These dynamic properties of PIn have been reported in mediated based biofuel cell due to its good electrocatalytic activity^40^.

**Figure 1:**
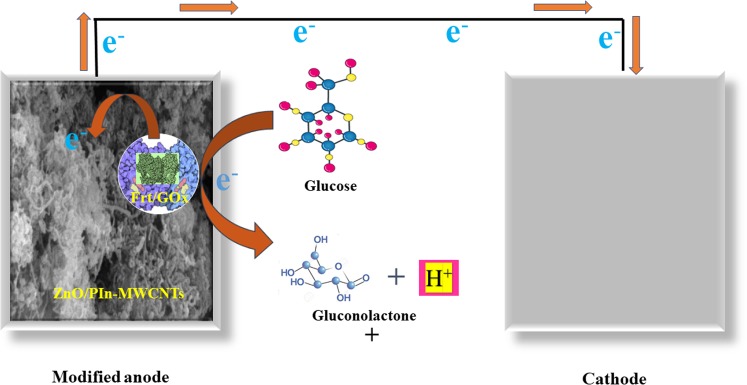
The assembly of bioanode and the shuttling of electrons via a biochemical pathway.

Here, in this work, efforts are made towards the development of polyindole functionalized-CNTs decorated by ZnO NPs as a scaffold for the wiring of GOx enzyme and its corresponding mediators. To the best of our knowledge, we inculcate the characteristics of individual component at one platform that reflect the synergistic properties which are favorable in selecting their counterparts and also to the best of our vision this is the first attempt that utilized ZnO-PIn doped CNTs based nanocomposite as a supporting material in developing bioanode for the wiring of enzyme in EBFCs application. The assembly of fabricated bioanode is schematically represented in Fig. 1.

## Experiment

### Materials used

Azadirachta indica Neem leaves obtained from the department of chemistry A.M.U Aligarh. Zinc nitrate hexahydrate, sodium hydroxide (NaOH), and double-distilled ethanol were purchased from Fisher Scientific, India. N-methyl pyrrolidone (NMP), phosphate buffers of pH 5.0 and 7.4 were procured from Alfa Aesar, India. Iron (III) chloride hexahydrate (FeCl_3_·6H_2_O) and sodium dodecylbenzene sulfonate (SDBS) were purchased from Central Drug House, Pvt. Ltd. India. Indole monomer, ferritin (10 mg ml^−1^ in 0.15 M NaCl), 2% aqueous solution of glutaraldehyde and glucose oxidase (GOx) obtained by Aspergillus niger were purchased from Sigma-Aldrich, India. All chemicals were used without further purification. Double distilled water (DDW) was utilized for washing purposes.

### Instruments used

The tropical and in-depth evidence of the synthesized ZnO/PIn-MWCNTs nanocomposite was manifested by scanning electron microscopy (SEM) (JSM, 6510 LV, JEOL, Japan) and transmission electron microscopy (TEM) (TEM 2100, JEOL, Japan) that operated at 200 kV on a carbon-coated copper grid. Moreover, elemental dispersive X-ray (EDX) reveals the elemental composition. Furthermore, the phase of the crystal and the functional groups were determined via X-ray diffraction (XRD) analyses and Fourier transform infrared spectroscopy (FTIR), respectively. The three-electrode system, such as modified glassy carbon electrode (GCE), Pt wire, and Ag/AgCl (3 M KCl), utilized as working, counter and reference electrode, respectively, that all are integrated with the potentiostat/galvanostat (PGSTAT 302 N Autolab, Switzerland) for electrochemical assessment of modified electrode.

### Synthesis of ZnO NPs by using neem leaves extract

The Neem (A. indica) leaves were collected and washed thoroughly with tap water to remove debris and other contaminated organic contents, followed by double distilled water and air-dried at room temperature. Finely cut leaves were kept in a beaker containing double distilled water and boiled for 30 min. The extract was cooled down and filtered with Whatman filter paper no.1, and the extract was stored at 4 °C after covering the beaker with aluminium foil for further use.

Firstly, 10 mL of neem extract was mixed with 90 mL of 0.1 M of zinc nitrate solution drop-wise under continuous agitation at ambient temperature for 4–5 h. After that, 50 mL of 2.0 M NaOH solution was added drop-wise in to the above mixture and allowed the mixture for 2 h continuous stirring. The resulting white precipitate was filtered and washed repeatedly with distilled water followed by ethanol to remove the impurities. Finally, a white powder was obtained after overnight drying of the purified precipitate at 60 °C in an oven. The dried ZnO NPs were scrapped out for further analysis^33^.

### Preparation of (ZnO/PIn-MWCNTs) nanocomposite

**Figure 2:**
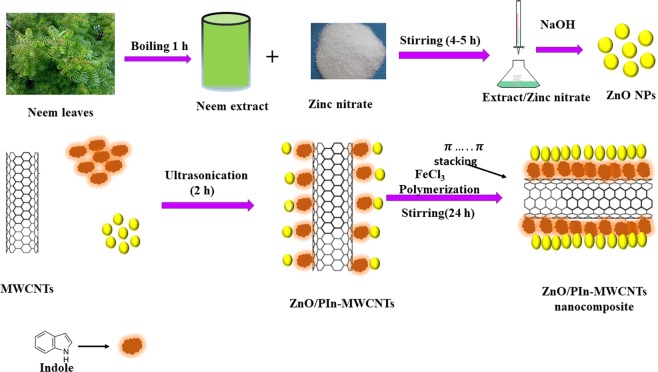
Schematic illustration of steps involved in preparation of (ZnO/PIn-MWCNTs) nanocomposite.

The ZnO/PIn-MWCNTs nanocomposite was synthesized by using the *in-situ* chemical oxidative polymerization of the indole monomer over the surface of MWCNTs, as shown in Fig. 2. Firstly, 10 mg of pristine MWCNTs mixed with 8 mmol of SDS in 20 mL DMW and kept the mixture in the ultrasonication bath for 2 hr. Simultaneously, 20 mg of ZnO NPs in 20 mL DMW, as prepared above, was taken in a separate beaker and placed inside the ultrasonication bath for the same period. After that, 26 mg of indole monomer added to this suspension and allowed the suspension for continuous mixing up to 2 hr. After ultrasonication, transfer the mixture on to the magnetic stirring and immediately added 40 mL of a 0.4 M FeCl_3_ solution into it and left the mixture to complete polymerization reaction for 24 hr via continuous stirring. During this, the color of the mixture turned to dark black and the solid product obtained through centrifugation after three times washing with DMW or till the filtrate appear colorless. Lastly, the solid product dried in a vacuum oven at 60 °C for 24 hr and converted into fine powder by mortar and pestle for further analysis^27^.

### Preparation of (ZnO/PIn-MWCNTs) nanocomposite dispersion

The suspension of ZnO/PIn-MWCNTs nanocomposite was obtained by solvating 10 mg of the prepared product in 5 mL NMP solvent. The performance of scattering was checked by a UV-vis spectrophotometer between the range 300–700 nm.

### Preparation of ZnO/PIn-MWCNTs/Frt/GOx bioanode

Firstly, a glassy carbon electrode (GCE) of diameter 3 mm was cleaned by alumina slurry taken on a velvet pad. Then, the electrode was sonicated in DDW and ethanol for 10 min successively. In a nutshell, a 6 µL of the prepared catalytic ink of nanocomposite was cast on the dry surface of GCE. After 4 hr of air dry, 5 µL of Frt mediator was dropped on the dried surface of the nanocomposite. After that, 6 µL of GOx enzyme solution prepared in PBS of pH 5.0 of concentration 10 mg/ml was deposited on the previously dried coating. Lastly, 1.5 µL glutaraldehyde (2% aqueous solution) was applied over the dry coating of the enzyme for maintanace the strong cross-linking in the GOx enzyme by avoiding the leakage problem. After 30 min of air-drying the developed electrode was dipped into the DDW for a while to exculpate the untied enzyme and put it inside the refrigerator at 4 °C when it was not in use.

## Results and Discussion

### Characterization

**Figure 3:**
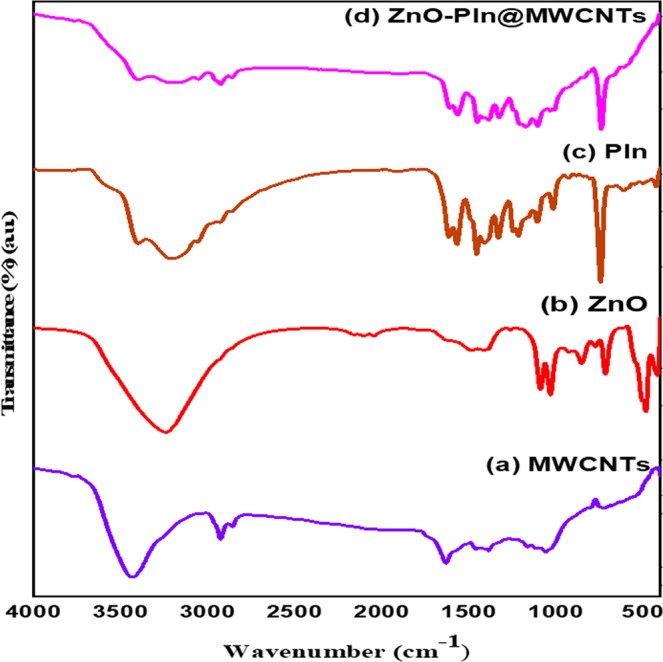
FTIR spectra of (**a**) MWCNTs (**b**) ZnO NPs (**c**) PIn (**d**) the synthesized nanocomposite (ZnO/PIn-MWCNTs).

Fourier transform infrared (FTIR) spectroscopy was employed for the identification of the structure of the synthesized ZnO/PIn-MWCNTs nanocomposite by comparing the spectra of individual counterparts, as displayed in Fig. 3. Figure 3(a) reflects the vibrations of MWCNTs at 3400 cm^−1^, 2917 cm^−1^, and 1632 cm^−1^, which correspond to the O-H, C-H, and C=O stretching vibrations, respectively^41^. The spectrum manifests the O-H vibration is due to the presence of moisture at the surface of CNTs, C-H vibration is because of sp^2^ carbon of the ring, and C=O vibration is due to the availability of carbonyl group owing to some oxidize carbon. The biologically synthesized spectrum of ZnO is shown in Fig. 3(b). This represents the peaks around 488 to 716 cm^−1^ owing to the vibration of the Zn-O bond that confirms the presence of M-O vibrations. Whereas the vibration corresponding to 848 cm^−1^,1026 cm^−1^, 1096 cm^−1^, and 3228 cm^−1^ reflect the vibrations of C–H stretching of aliphatic alkane chain (neem extracts), C–N stretching vibrations of aliphatic, aromatic amides and O-H stretching of intramolecular H-bond, respectively^33^. Furthermore, the spectrum of PIn as shown in Fig. 3(c) displays the vibration at 734 cm^−1^ corresponding to the presence of C-H bond in the benzene ring of indole, the vibration at 1104 cm^−1^ due to stretching of C-N bond, whereas the bands at 1222 cm^−1^ and 1430 cm^−1^ are characteristics of the aromatic ring stretch vibrations in PIn^42^. It is to be noted that the spectrum of ZnO/PIn-MWCNTs nanocomposite given in Fig. 3(d) manifested all the bands as found in the individual spectrum with less peak intensity. It can be concluded that the components of nanocomposite exhibit strong interaction with their counterparts.

**Figure 4:**
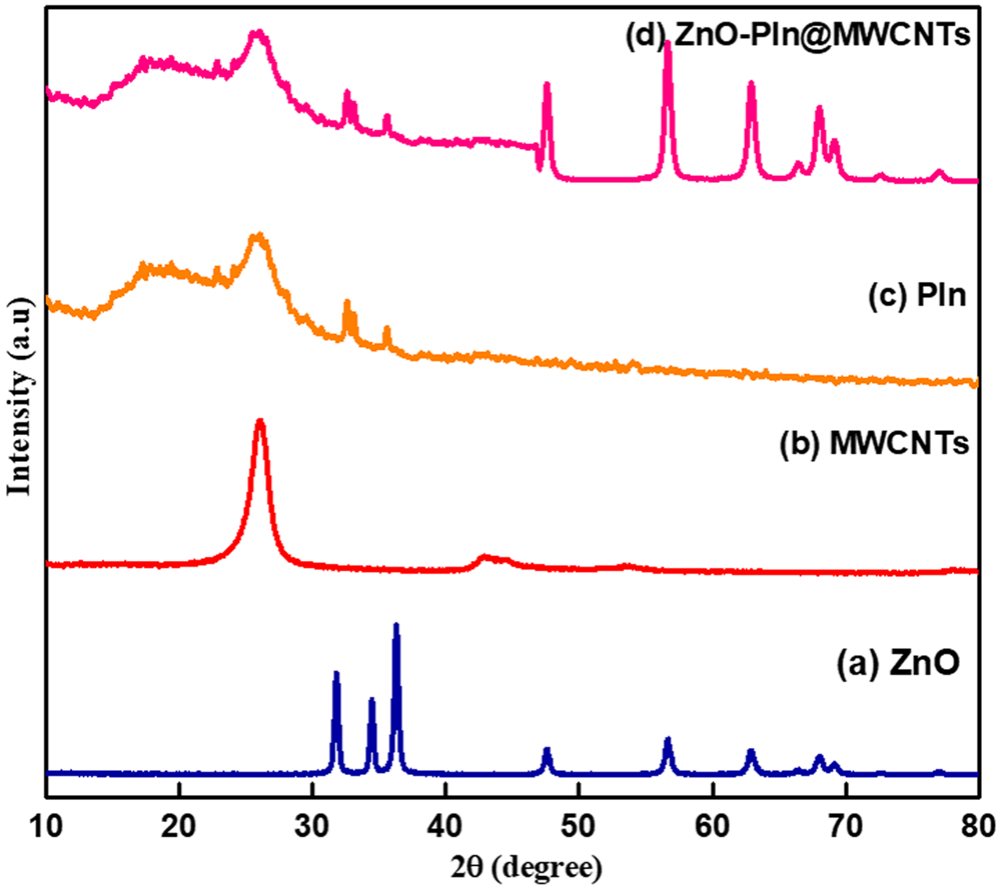
XRD pattern of (**a**) ZnO NPs (**b**) MWCNTs (**c**) PIn (**d**) the synthesized nanocomposite (ZnO/PIn-MWCNTs).

The XRD pattern of ZnO NPs, MWCNTs, PIn, and the synthesized ZnO/PIn-MWCNTs nanocomposite were recorded and the results obtained are manifested in Fig. 4. The 2θ pattern of biologically synthesized ZnO NPs **(curve a)** shows the characteristic peaks at 31.66°, 34.58°, 36.54°, 47.76°, 56.5°, 62.7°, 66.32°, 67.76°, and 69.18°^33^. These reflection lines corresponding to the wurtzite structure that confirmed the successful synthesis of ZnO NPs. The diffraction pattern of **curve b** observed at 26.1° and 43.8° is well-matched with the hexagonal graphitic structure of CNTs^18^ whereas the spectrum of **curve c** reflects the broad peaks around 15–21.4°, 26°, and 33° that manifested the amorphous phase of PIn^43^. Additionally, the spectrum of ZnO/PIn-MWCNTs nanocomposite as shown in the **curve d**, exhibit almost all the diffraction patterns as found in the counterparts well explained above. Hence, the XRD analysis reveals the successful synthesis of ZnO/PIn-MWCNTs nanocomposite.

**Figure 5:**
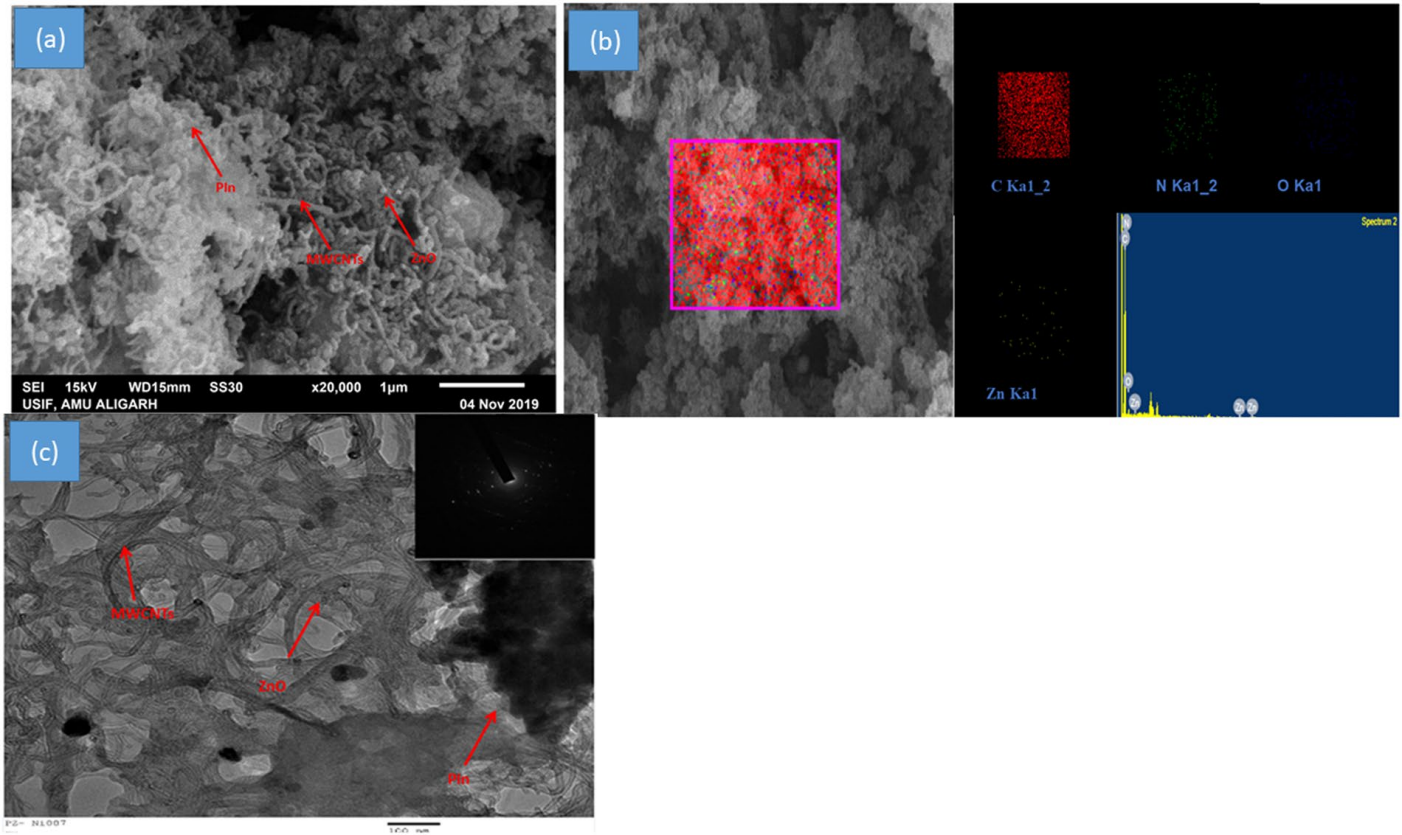
SEM image of (**a**) ZnO/PIn-MWCNTs nanocomposite (**b**) EDX mapping of the synthesized nanocomposite (ZnO/PIn-MWCNTs); (**c**) TEM image of nanocomposite (ZnO/PIn-MWCNTs) with the inset of SAED.

The surface morphology and elemental composition of *in-situ* oxidative polymerization of indole based ZnO/PIn-MWCNTs nanocomposite, are shown in Fig. 5(a,b). The SEM micrograph, as shown in **curve a** reveals the entangled assembly of MWCNTs in the matrix. Furthermore, the image shows the spherical morphology of ZnO NPs that are uniformly scattered over the surface of PIn-MWCNTs. The PIn-MWCNTs display the strong interaction among each other which is due to the existence of Π- Π stacking that leads to compact entangle morphology. Besides, the nitrogen-containing PIn group attracts the ZnO NPs towards it that leads to a uniform distribution over the surface of PIn-MWCNTs. The micrograph shows the porosity in their structure which improves the loading of the enzyme by serving as host for the efficient binding of GOx and its mediator. Hence, it is deep-rooted that the synthesized ZnO/PIn-MWCNTs nanocomposite work as good supporting material for the wiring of the enzyme. The elemental composition of developed material such as carbon, oxygen, nitrogen, and zinc confirmed by EDX mapping can be seen in figure b.

Moreover, the TEM micrograph, as shown in Fig. 5(c) demonstrate the highly uniform distribution of MWCNTs in the matrix of polyindole that are further decorated by ZnO NPs. The image confirms the intimate connection between the counterparts of the synthesized nanocomposite. Along with high porosity and a very large specific surface area due to the availability of homogenously dispersed CNTs and ZnO NPs that in turn, facilitate the electrical communication. Furthermore, the SAED (selected area electron diffraction) pattern shown in the inset of curve c represents concentric rings, confirmed the crystallinity of the synthesized nanocomposite which are further beneficial for the high enzyme loading^44^.

### Electrochemical studies

**Figure 6:**
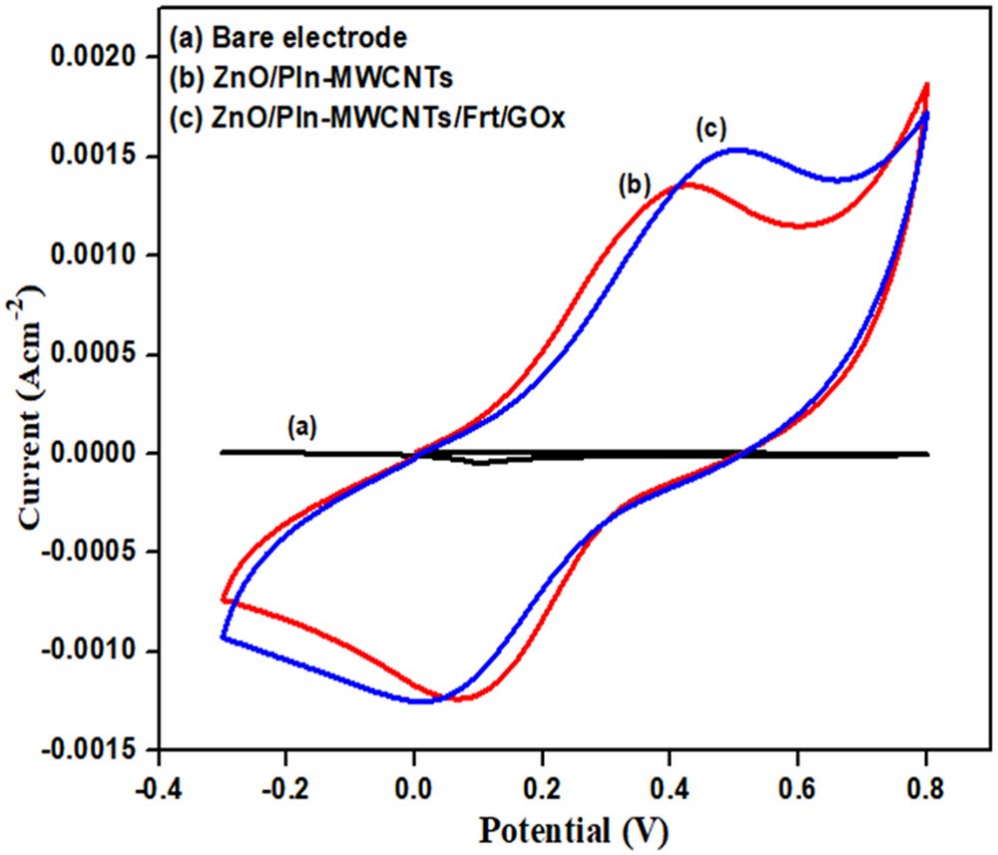
Cyclic voltagramms of (**a**) bare GCE (**b**) ZnO/PIn-MWCNTs (**c**) GCE ZnO/PIn-MWCNTs /Frt/GOx in PBS pH 7.4 as supporting electrolyte at ambient conditions.

The cyclic voltammetry (CV) study of the synthesized nanocomposite ZnO/PIn-MWCNTs and the modified ZnO/PIn-MWCNTs/Frt/GOx bioanode was carried out in PBS of pH 7.4, using as a supporting electrolyte. The cyclic voltammograms were recorded as given in Fig. 6(a) bare GCE electrode **(b)** ZnO/PIn-MWCNTs **(c)** ZnO/PIn-MWCNTs/Frt/GOx.

From the given outcomes, it can be seen that the bare GCE is in active towards the catalytic reaction, whereas a well-defined redox peak was found in the case of GCE electrode modified with ZnO/PIn-MWCNTs nanocomposite. Moreover, the amplification in peaks was observed in the case of complete bioanode (ZnO/PIn-MWCNTs/Frt/GOx). From the given outcomes, it can be concluded that the catalytic peaks were observed due to the presence of highly conducting MWCNTs that have a very high surface area which permits the flow of electrons and hence improve the catalytic activity. Furthermore, CNTs are wrapped by PIn redox polymer anchored with ZnO NPs. They both contribute to the enhancement of electrochemical performance. It inferred that the increase in current is due to the strong covalent interaction of nitrogen atom of PIn with the ZnO nanoparticles along with the π-π stacking exhibits between the π ring of PIn and an enclosed ring of MWCNTs. The components of ternary nanocomposite are redox-active that shows the oxidation peak current of 1.34 mA cm^−2^ when the circuit is complete. Moreover, the increase in current density of 1.528 mA cm^−2^ was observed after the immobilization of Frt and GOx with a couple of redox peak at 0.4 V and 0.03 V, this upward shift in the peaks reveal that the ZnO/PIn-MWCNTs nanocomposite has the porous network structure which allows the entrapping sites for the loading of GOx thereby significantly amplifying the active surface area of the electrode. This inflation in current favors the successful binding of the enzyme on to the surface of the synthesized material.

**Figure 7:**
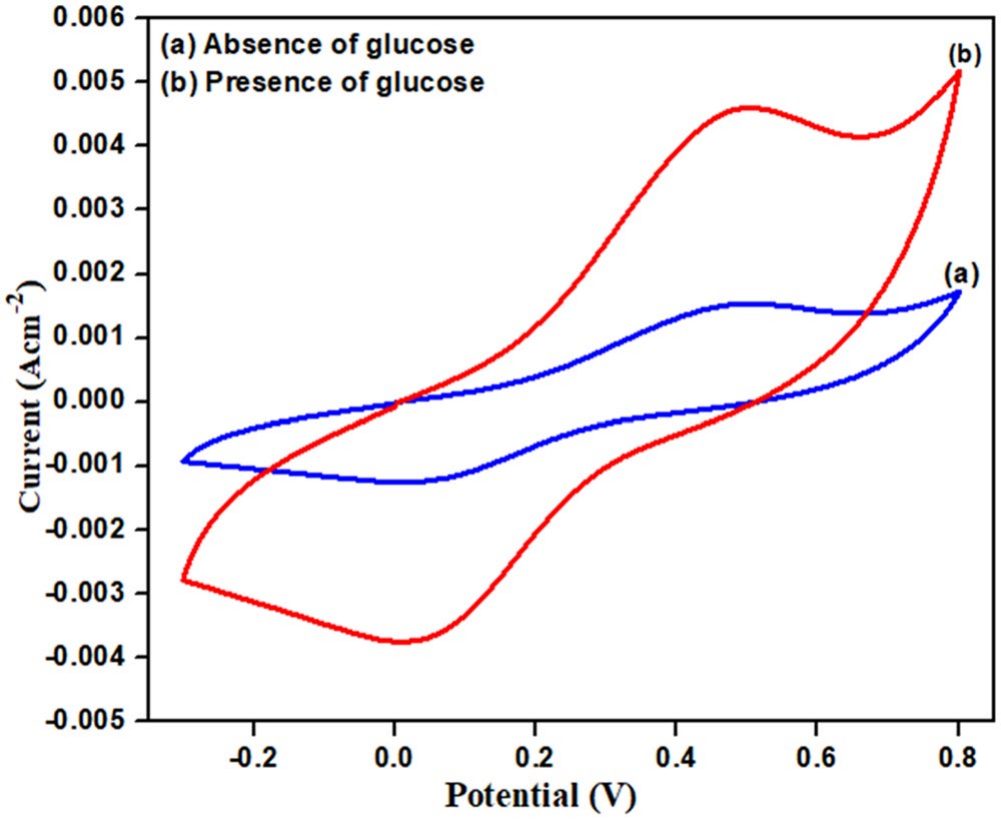
Cyclic voltagramms of GCE/ ZnO/PIn-MWCNTs /Frt/GOx. (**a**) in absence of glucose and (**b**) in presence of 50 mM glucose concentration.

The bio-electrocatalytic performance of fabricated ZnO/PIn-MWCNTs/Frt/GOx electrode was assessed by the addition of 50 mM glucose in the PBS as supporting electrolyte that shows the rapid increase in catalytic activity by delivering the current density of 4.9 mA cm^−2^ as shown in Fig. 7(b). This value is large in magnitude than the fabricated ZnO/PIn-MWCNTs/Frt/GOx electrode when observed in the absence of glucose as can be seen in Fig. 7(a). The increase in biocatalytic activity is due to the splitting of glucose into gluconolactone thereby releasing the electrons which enhance the catalytic activity. It can be concluded that the synthesized nanocomposite is a good supporting material for the wiring of enzyme and its mediator and possess excellent electron transfer ability.

**Figure 8:**
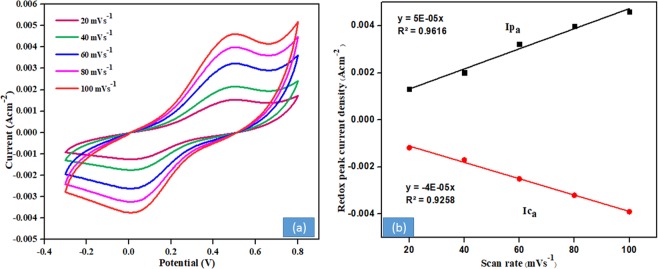
(**a**) Cyclic voltagramm of GCE/ ZnO/PIn-MWCNTs /Frt/GOx at different scan rate ranging from 20–100 mV s^−1^ in presence of 50 mM glucose. (**b**) redox peaks calibration curve of the respective bioanode by varying the scan rate from 20–100 mV s^−1^.

Interestingly, it can be seen that the redox peaks current sharply increases by varying the applied potential from 20–100 mV s^−1^, as shown in Fig. 8(a). It can be attributed to the good electrocatalytic activity of the MWCNTs doped with polyindole and the high catalytic activity of ZnO NPs which accelerate the electron shuttling. Therefore, an increase in catalytic activity is a result of the highly conducting components of bioanode. Also, this enhancement signifies that the reactions occurring on the ZnO/PIn-MWCNTs/Frt/GOx fabricated electrode were quasi-reversible surface-controlled reaction. On the other side Fig. 8(b) displays the respective calibration curve of redox peak current against the scan rate that delivers the linear relation along with the regression equations of *lpa* = 0.00004*x* + 0.0005, *lpc* = −0.0003*x* − 0.0004 and 0.9616 and 0.9258 their correlation coefficients, respectively.

Besides, to illustrate the kinetics of the ZnO/PIn-MWCNTs/Frt/GOx modified bioanode, a prominent Laviron equation was used as given in equation [1]. The rate constant (k_s_) of the fabricated bioanode was calculated to be 4.28 s^−1^ at 100 mV s^−1^.

1 $$log{k}_{s}=\alpha \,\log (1-\alpha )+(1-\alpha )log\alpha -\,\log \left(\frac{RT}{nFv}\right)-\alpha (1-\alpha )\left(\frac{nF\Delta Ep}{2.3RT}\right)$$

Here, the symbols have their specific meaning such as *α*, *R*, *T*, *F*, *n*, *v* and ΔEP = Epa-Epc, represent the charge transfer coefficient, gas constant, temperature, Faraday’s constant (96485 Cmol^−1^) number of electron transfer, scan rate (100 mVs^−1^) and difference in peak potential respectively.

Besides, well known Brown-Anson model was utilized for the estimation of surface concentration of the enzyme immobilized on ZnO/PIn-MWCNTs/Frt/GOx bioanode which is calculated to be 3.4 × 10^−7^ mol cm^−2^, as given in Eq. (1) elsewhere^45^.

### EIS study

**Figure 9:**
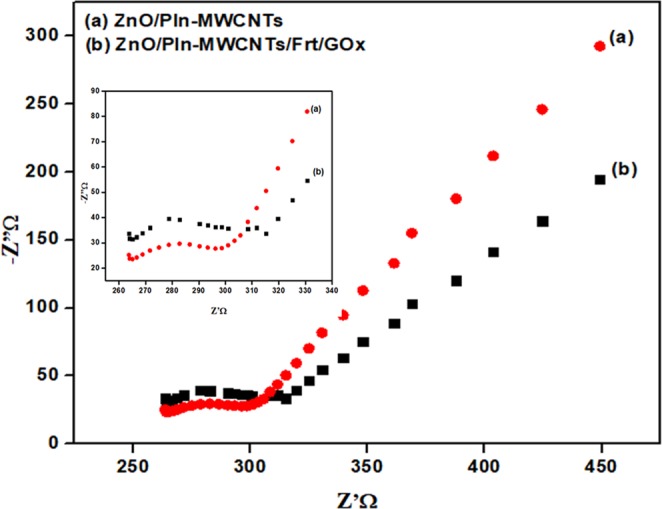
Nyquist plot of (**a**) GCE/ ZnO/PIn-MWCNTs (**b**) GCE/ ZnO/PIn-MWCNTs/Frt/GOx.

Furthermore, the charge-transfer resistance (R_**ct**_) of the developed ZnO/PIn-MWCNTs/Frt/GOx bioanode and the ZnO/PIn-MWCNTs supporting nanocomposite was investigated by the Nyquist plot obtained through EIS study. As can be seen in Fig. 9, the ZnO/PIn-MWCNTs nanocomposite **(curve a)** has a small semicircle (R_**ct**_, 37 Ω) compare to that of ZnO/PIn-MWCNTs/Frt/GOx modified bioanode (R_**ct**_, 51 Ω). From the given outcomes, it can be concluded that the small diameter of the curve demonstrating the good electron transfer ability to conduct nanocomposite while the elevation in **curve b** was observed after immobilization of Frt and GOx which suggests the delay in electrical communication. This increase in resistance is due to the encapsulation of enzyme and its mediator by a protein shell which hindered the electron shuttling and simultaneously, this result affirmed the successful wiring of Frt and GOx on the supporting materials.

### LSV analysis

**Figure 10:**
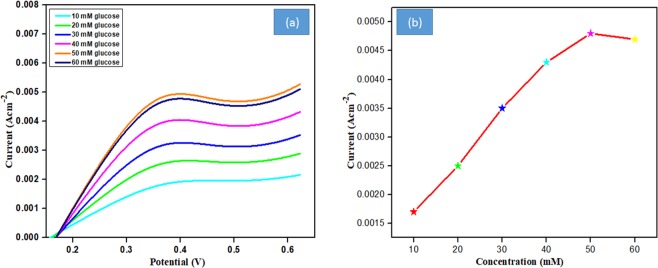
(**a**) Linear sweep voltammogram of GCE/ ZnO/PIn-MWCNTs/Frt/GOx by increasing glucose range (10–60 mM) in PBS pH 7.4 at ambient temperature. (**b**) calibration curve plotted against the anodic current versus glucose concentration.

The optimization of the electrocatalytic activity of the developed ZnO/PIn-MWCNTs/Frt/GOx bioanode was assessed by varying the concentration of glucose from 10–60 mM. The linear sweep voltammetry (LSV) study reveals the response of catalytic activity towards glucose concentration. As can be seen in Fig. 10(a), there is a gradual increase in oxidation peak height along with the rise in the substrate (glucose) up to 50 mM concentration. Then after, the decrease in peak height was noticed. This outcome suggested that the synthesized ZnO/PIn-MWCNTs nanocomposite has enough binding sites due to its porous network structure which increases the loading of enzyme and hence, catalytic activity.

Further decrease in the peak was observed which suggests the saturation kinetics of the reaction. It also affirmed the filling of all the empty voids of the nano-biocatalyst and hence not affected by a further increase in concentration. Moreover, the optimized current density obtained through bioelectrocatalysis of glucose is 4.9 mA cm^−2^ at a scan rate of 100 mV s^−1^ by consuming 50 mM glucose in PBS of pH 7.4 can be seen in calibration **curve b** plotted against the anodic current versus glucose concentration.

## Conclusion

The ternary nanocomposite comprising of PIn, MWCNTs, and ZnO NPs was successfully synthesized via the *in-situ* chemical oxidative polymerization route. Here, we have integrate the captivating properties of glucose oxidase with MWCNTs based electrodes by modifying through electrostatic adsorption of GOx on the electrode surface, modified by the ZnO NPs decorated on the conducting polyindole. The synthesized ZnO/PIn-MWCNTs nanocomposite offer a suitable microenvironment to retain its catalytic activity, which accelerates the electron shuttling from the enzymes to the electrodes. The prepared material exhibited excellent performance attributed to synergistic effects among the catalytic activity of ZnO NPs and the superior conductivity of PIn doped CNTs. It is anticipated that the ZnO/PIn-MWCNTs hybrid nanocomposite might be a promising candidate for assembling electrochemical biofuel cells as well as in biosensors.
